# Pancreatic Paraganglioma: A Case Report

**DOI:** 10.1089/crpc.2016.0016

**Published:** 2016-12-01

**Authors:** Sumant Tumuluru, Vincent Mellnick, Maria Doyle, Bella Goyal

**Affiliations:** ^1^ Mallinckrodt Institute of Radiology, Washington University in St. Louis, St. Louis, Missouri.; ^2^ Department of Surgery, Washington University in St. Louis, St. Louis, Missouri.; ^3^ Department of Pathology & Immunology, Washington University in St. Louis, St. Louis, Missouri.

**Keywords:** paraganglioma, pancreas, extra-adrenal

## Abstract

**Background:** Paraganglionic neoplasms that originate in the adrenal medullas are referred to as pheochromocytomas, but if they arise from other paraganglia scattered throughout the body, they are referred to as paragangliomas. Pancreatic paragangliomas are an extremely rare entity as only 20 cases have been reported in the literature. They tend to be nonfunctional and typically occur in the fourth to fifth decade of life without a gender predilection. We describe in this study a case of a pancreatic paraganglioma and its CT appearance.

**Case Presentation:** A 62-year-old woman undergoing presurgical evaluation for an olfactory groove meningioma resection was incidentally found to have a pancreatic mass. Multiple fine needle aspirations of the mass through endoscopic ultrasound yielded only atypical epithelial cells. The mass demonstrated avid enhancement on serial CTs with mild interval growth over a period of 5 years. No lymphadenopathy was ever found. The patient's complete blood count, complete metabolic panel, and plasma carcinoembryonic antigen levels were all within normal limits. Urine catecholamine metabolite levels were never checked as the patient demonstrated no symptoms of catecholamine excess. The patient underwent a laparoscopic distal pancreatectomy and splenectomy, and the mass was eventually diagnosed as a pancreatic paraganglioma through pathology. While the patient tolerated the surgery well, she did require a biliary sphincterotomy and placement of a pancreatic duct stent postoperatively for treatment of a pancreatic duct leak, which completely resolved. She showed no evidence of disease recurrence on multiple subsequent CTs and continues to do well.

**Conclusion:** Pancreatic paragangliomas are usually incidentally discovered and typically demonstrate avid homogenous enhancement on contrast-enhanced CT or MR. Aggressive surgical resection is necessary to maximize the chances of disease-free survival. Pancreatic paragangliomas are similar histologically, whether benign or malignant, to paragangliomas that occur anywhere else in the body, with ∼70% in the abdomen and 30% in the chest.

## Introduction and Background

Paraganglia are specialized cells derived from the neural crest that lie in close proximity to sympathetic ganglia throughout the body. Components of the paraganglionic system include the adrenal medulla, the carotid, aortic and vagal bodies, and scattered ganglia throughout the chest and abdomen.^1^ They are involved with producing rapid physiologic changes in the body through the production of catecholamines.^2^ Tumors originating in the adrenal medulla are referred to as pheochromocytomas, whereas if they arise from other paraganglia, they are referred to as paragangliomas. Their incidence is equal in both men and women, and they typically occur during the fourth to fifth decade of life.^1^ These tumors can be functional and produce excess catecholamines in more than half the cases. Hence, common presenting symptoms include palpitations, headache, sweating, and hypertension.^2^ Retroperitoneal paragangliomas are rather uncommon, but paragangliomas arising within the pancreas are exceedingly rare as only 20 cases have been reported in the literature.^3^ Pancreatic paragangliomas tend to be nonfunctional, which is in contrast to paragangliomas that occur in other more common locations. In contrast to pancreatic adenocarcinomas, pancreatic paragangliomas do not typically cause biliary obstruction.^4^ Hence, they present as either incidental findings on imaging or in a patient with abdominal pain and/or a palpable abdominal mass.^5^ We describe in this study a case of a pancreatic paraganglioma.

## Case Presentation

**FIG. 1.**
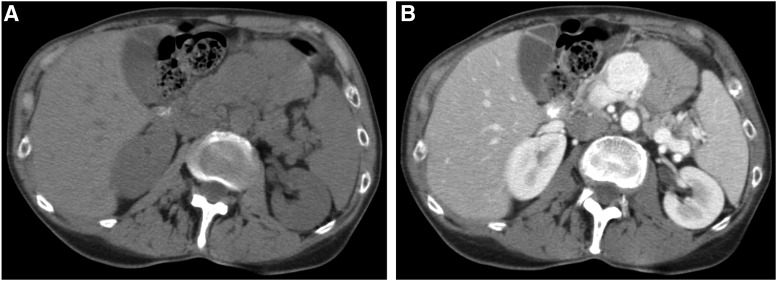
**(A)** Noncontrast transaxial CT demonstrates a discrete round mass in the pancreatic body. **(B)** Contrast-enhanced transaxial CT demonstrates avid homogeneous enhancement of the mass.

**FIG. 2.**
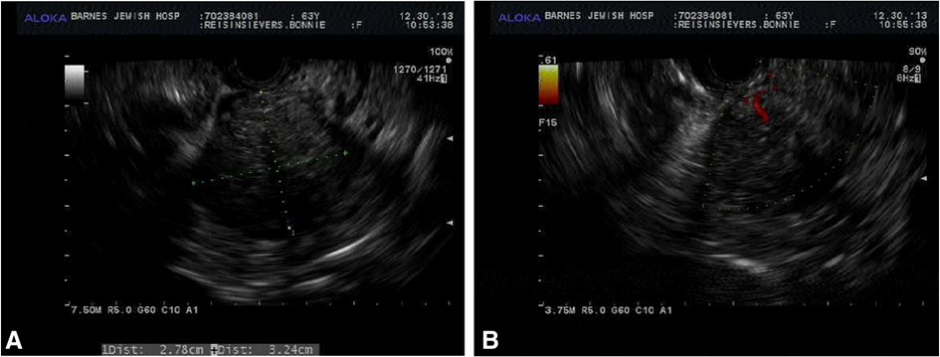
**(A)** Endoscopic ultrasound demonstrates a 3.2 × 2.8 cm hypoechoic discrete mass in the pancreatic body. **(B)** Color Doppler through endoscopic ultrasound demonstrates internal vascularity of the mass.

A 62-year-old woman undergoing presurgical evaluation for an olfactory groove meningioma resection was incidentally found to have a pancreatic mass on an abdominal CT in April 2009. The 2.9 × 2.5 cm mass was centered in the pancreatic body with a round shape, well-defined margins, and avid homogenous enhancement (Fig. 1A, B). There was mild upstream pancreatic duct dilation. There was no lymphadenopathy in the abdomen and pelvis or other signs of another primary tumor or metastatic disease. Differential considerations offered included either a neuroendocrine tumor or a vascular abnormality such as an aneurysm or pseudoaneurysm. An endoscopic ultrasound performed at the time revealed a 2.2 cm well-defined solid hypoechoic mass in the pancreatic body, suspicious for a pancreatic neuroendocrine tumor. Fine needle aspiration of this mass showed no evidence of a malignancy, but only changes of chronic pancreatitis. The patient's abdominal physical examination was normal, and she denied any abdominal pain, weight loss, diarrhea, or constipation. Repeat CT in October 2013 demonstrated mild interval increase in size of the mass to 3.0 × 3.0 cm. An endoscopic ultrasound was repeated in December 2013, confirming an increase in size of the solid mass (Fig. 2A, B). Repeat fine needle aspiration of the mass yielded only atypical epithelial cells. The patient's complete blood count, complete metabolic panel, and plasma carcinoembryonic antigen levels were all within normal limits. Urine catecholamine metabolite levels were never checked presurgically as the patient exhibited no symptoms or signs of excess catecholamine production. Hence, metaiodobenzylguanidine (MIBG) scintigraphy was also not performed.

The patient underwent a laparoscopic distal pancreatectomy and splenectomy in March 2014 for excision of the mass. A well-circumscribed tan mass measuring 2.8 × 2.8 × 2.7 cm was found on the posterior aspect of the pancreas and was diagnosed as a paraganglioma through pathology. The rest of the pancreas and spleen were unremarkable. The patient tolerated the procedure well, but developed a pancreatic duct leak after the surgery. This was treated with a biliary sphincterotomy and placement of a pancreatic duct stent, which led to complete resolution of the leak. Plasma metanephrine and normetanephrine levels when checked 2 weeks status postsurgery were within normal limits. Multiple repeat CT scans since the surgery, with the most recent one in September 2015, showed no evidence of recurrent disease or lymphadenopathy, and the patient continues to do well.

## Discussion

While paragangliomas can occur anywhere, there is paraganglionic tissue in a para-aortic distribution; they are most common in the infrarenal region where the organ of Zuckerkandl is present. Some uncommon locations include the gallbladder, urinary bladder, prostate, spermatic cord, uterus, and duodenum.^2^ Approximately, 70% of the sympathetic paragangliomas occur in the abdomen, whereas the other 30% occur in the chest. Paragangliomas can metastasize in ∼20–42% of reported cases.^6^ The most common sites of metastases include regional lymph nodes, bone, lung, and liver. The pathway for metastases can be either hematogenous or lymphatic spread. As both benign and malignant paragangliomas have the same imaging and histological appearance, the best prognostic indicators are the presence of metastases and disease recurrence.^1^ Currently, there are no genetic, molecular, or imaging markers that can accurately and reliably identify malignant potential.^3^

On contrast-enhanced CT, paragangliomas appear as soft tissue masses with either avid homogenous enhancement or with central areas of hypoattenuation. Smaller paragangliomas are more likely to be homogenously enhancing and more discrete.^2^ As was the case with our patient, small paragangliomas can look remarkably similar to a neuroendocrine tumor or a vascular lesion such as an aneurysm or pseudoaneurysm on CT. This necessitates further imaging with either MR or ultrasonography. They can also be predominantly cystic, especially if they are large.^7^ A majority of the previously reported pancreatic paragangliomas were located in the head of the pancreas.^8^ However, they do not cause biliary or pancreatic ductal dilatation or obstruction.^9^ Our case was a bit abnormal as there was mild upstream pancreatic duct dilatation. However, it wasn't a functional obstruction, and there were no laboratory abnormalities. On MR, paragangliomas are either hypointense or isointense to liver parenchyma on T1-weighted images and are substantially hyperintense on T2-weighted images.^2^ Sometimes, they can contain fluid-fluid levels. On ultrasonography, they appear as well-demarcated masses with hypoechoic areas.^10^ As they are hypervascular, an intense blush is seen within the mass secondary to enlarged feeding arteries on catheter angiography. Despite the known appearance of paragangliomas on multiple imaging modalities, MR with intravenous contrast is often preferred due to its superior soft tissue contrast resolution. Anatomic imaging, however, cannot differentiate between functioning and nonfunctioning paragangliomas. Functional paragangliomas can be detected through the use of MIBG scintigraphy. Once MIBG is tagged with either ^123^I or ^131^I, it can be taken up by functional paragangliomas. This makes the detection of functional paragangliomas easier, particularly when they are in atypical locations, multiple in number, or metastatic. While MIBG scintigraphy has excellent specificity (95–100%), its sensitivity is slightly lower at 85%. Moreover, as it has a false negative rate around 10–15%, anatomic imaging with CT or MRI is usually performed as an adjunct.^2^

**FIG. 3.**
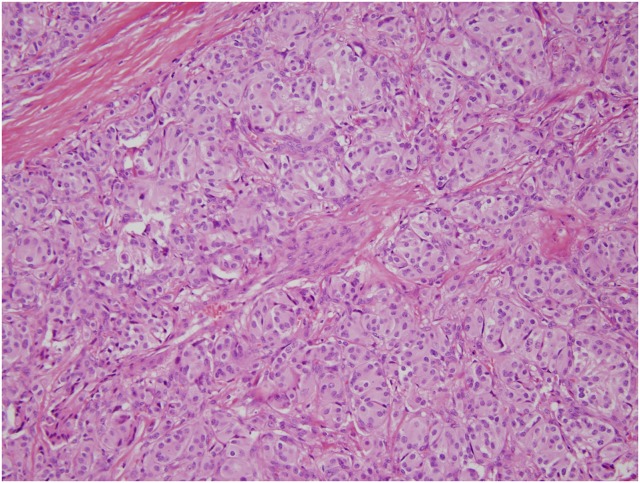
Hematoxylin and eosin stain of a pancreatic paraganglioma; 200 × magnification. There is a typical “zellballen” pattern, or small nests, of polygonal cells with prominent nucleoli and abundant finely granular eosinophilic to amphophilic cytoplasm. The nests are surrounded by a delicate fibrovascular network.

All paragangliomas are similar histologically regardless of their site of origin or their malignant potential. They contain clusters of cells in a nesting or trabecular pattern separated by a highly vascular reticular network (Fig. 3). These clusters can be composed of either round or oval cells or giant multinucleated cells. The cells contain abundant homogenous or finely granular cytoplasm that can be either clear or eosinophilic. They may demonstrate nuclear atypia or vascular invasion and are usually not necrotic. The nuclei tend to be large and normochromatic, with occasional irregular and hyperchromatic forms.^11^ Immunohistochemistry usually shows these tumors to be positive for synaptophysin and chromogranin.^12^ Features suggestive of malignancy include a high mitotic index and a low reactivity for neuropeptides by immunohistochemistry, but these have not been reliably verified.^13^

A correct diagnosis of a pancreatic paraganglioma is difficult to make preoperatively. They will often be misdiagnosed as either neuroendocrine tumors or potentially pancreatic cystic lesions given their similar appearance and lack of characteristic symptoms.^14^ Therefore, a pathological diagnosis is necessary for confirmation. While fine needle aspiration can be performed, it is frequently either inaccurate or nondiagnostic.^3^ Surgical excision is the main treatment of choice as there exists a possibility of malignant transformation. For pancreatic head lesions, a pancreaticoduodenectomy is recommended, while a distal pancreatectomy is recommended for pancreatic body and tail lesions.^12^ Aggressive surgery is necessary to ensure disease-free survival. As was done in our patient, a CT of the chest, abdomen, and pelvis should be performed before surgical resection to rule out any metastases. In addition, routine continued postsurgical CT surveillance is necessary to ensure no recurrence of disease as recurrence is a hallmark of malignancy. Radiation therapy has also been used in patients who are not deemed operative candidates or in patients with malignant disease status postsurgical resection.

## Conclusion

Pancreatic paragangliomas are an exceedingly rare subset of retroperitoneal paragangliomas. They tend to be nonfunctional and present either incidentally on imaging, as a palpable abdominal mass, or in a patient with abdominal pain. When functional, they will cause the typical symptoms of excessive catecholamine release such as palpitations, headache, sweating, and hypertension. Given that our patient was asymptomatic, preoperative measurement of urine catecholamine metabolites was deemed unnecessary, but they can certainly be useful in the diagnosis of functional paragangliomas. They typically demonstrate avid homogenous enhancement on contrast-enhanced CT or MR. Small paragangliomas, as in our case, can look remarkably similar to pancreatic neuroendocrine tumors and vascular lesions. This necessitates imaging with multiple modalities to narrow the differential diagnosis. Ultimately, pathologic diagnosis is necessary. They are treated through aggressive surgical resection as the presence of metastases or disease recurrence is a poor prognostic indicator of survival.

## Abbreviations Used

CT: computed tomography
MIBG: metaiodobenzylguanidine
MRI: magnetic resonance imaging
